# Comparative Fractal Analysis on Orthopantomography Images of Bone Remodeling Changes at 3 Months Between Natural Socket Healing and Immediate Implant Placement

**DOI:** 10.3390/diagnostics16010096

**Published:** 2025-12-27

**Authors:** Andrei Radu, Mihaela Ionescu, Antonia Samia Khaddour, Cristina Maria Munteanu, Monica Mihaela Iacov Crăițoiu, Melania Olimpia Cojocaru, Alex Ioan Sălan, Iulia Roxana Marinescu, Răzvan Mercuț, Sanda Mihaela Popescu

**Affiliations:** 1 Department of Oral Rehabilitation, University of Medicine and Pharmacy of Craiova, 200349 Craiova, Romania; andrei.radu0692@gmail.com (A.R.); melania.cojocaru@umfcv.ro (M.O.C.); roxana.marinescu@umfcv.ro (I.R.M.); sanda.popescu@umfcv.ro (S.M.P.); 2 Department of Medical Informatics and Biostatistics, University of Medicine and Pharmacy of Craiova, 200349 Craiova, Romania; 3 Department of Oral and Maxillofacial Surgery, University of Medicine and Pharmacy of Craiova, 200349 Craiova, Romania; cristina.munteanu@umfcv.ro (C.M.M.); alex.salan@umfcv.ro (A.I.S.); 4 Department of Prosthetic Dentistry, University of Medicine and Pharmacy of Craiova, 200349 Craiova, Romania; monica.craitoiu@umfcv.ro; 5 Department of Plastic and Reconstructive Surgery, University of Medicine and Pharmacy of Craiova, 200349 Craiova, Romania; razvan.mercut@umfcv.ro

**Keywords:** molar, tooth extraction, fractal analysis, OPG, Image J, alveolar bone, implant-prosthetic therapy, bone regeneration

## Abstract

**Background:** Osseointegration is the main factor that ensures the long-term success of implant-prosthetic therapy, but besides this, there are other important factors, such as the quality of the alveolar bone and the time of placement of dental implants. The study aimed to analyze changes in the alveolar bone following tooth extraction, comparing natural healing with immediate implant placement, using fractal analysis on OPG images. **Methods:** This retrospective study included OPG images obtained before tooth extraction and 3 months after surgery in 91 patients who underwent maxillary and mandibular molar extractions and opted for either natural healing or immediate dental implant placement. Fractal analysis of OPG images was performed using Image J software, and the resulting measurements were subsequently statistically analyzed. **Results:** Most extractions were performed in the maxilla, and most were at the level of the first molar. The study group showed a faster healing process following immediate placement of dental implants, regardless of location, and a similar distribution of bone resorption and healing, with clear differences in location: the mandible had a faster healing process than the maxilla. **Conclusions:** Fractal analysis showed a better and quicker bone healing of the alveolar bone in immediate implant placement in molar areas compared with post-extraction natural healing, especially in the lower jaw.

## 1. Introduction

Alveolar bone has the property of remodeling throughout life [1], thus, following tooth extraction, dimensional changes inevitably occur in the alveolar bone, which may vary depending on the location of the extracted tooth [2,3,4,5], the extraction method [5], the quantity and quality of the remaining bone [6], as well as the patient’s health [7]. The success of implant treatment depends on several variables, including the type of dental implant, the surgical method, the quality of the alveolar bone, and the osseointegration process [8]. To ensure the success of implant-prosthetic treatment, it is essential to correctly assess the health of the jaw bones and peri-implant tissues [7,9,10]. The quality of the alveolar bone depends on the morphology and physiology of the bone, the degree of mineralization, as well as the trabecular pattern [11].

Implant-prosthetic treatment has become the standard for many clinical situations [12], with dental implants offering the advantage of maintaining the volume of the post-extraction alveolar bone and reducing bone resorption [13]. However, the success of implant treatment depends on uncomplicated surgical interventions, the healing and osseointegration periods, individual patient parameters, the type of dental implant, and the quality and quantity of bone tissue [7]. Brånemark defined osseointegration as the direct, functional connection between the alveolar bone and the surface of a dental implant [14]. To achieve osseointegration, variables such as the timing of implant placement, the macro- and microgeometry of the implant, and the condition of the alveolar bone must be considered [7,15]. In the literature, the timing of implant placement has been divided into three periods: immediate post-extraction placement, early placement, and late placement [16,17], each with its own advantages and disadvantages. Osseointegration protocols have indicated that dental implants should be placed in the mandible after 3–4 months of healing and in the maxilla after 6–8 months [7]. The advantages of immediate post-extraction dental implant placement include eliminating the waiting period for post-extraction socket healing, reducing the number of surgical interventions, shortening operating time, shortening the edentulous period, reducing cost, preserving post-extraction alveolar bone size, and reducing patient discomfort [12]. Early placement of dental implants (3–4 months of healing) represents a middle ground, allowing initial bone remodeling and stabilization while offering faster restoration compared to late placement. Late placement of dental implants (with more than 6 months of healing) is the conventional protocol, most commonly used in complex cases, with minimal risk of failure. Involves waiting for the alveolar bone to heal completely, but with the disadvantage of more advanced resorption [13,18].

Radiological evaluation of pre- and post-extraction bone tissue is an indispensable method for establishing a correct and successful implant-prosthetic treatment plan [10,19]. In current practice, various clinical and radiological examination methods are used, including periapical, panoramic, and cone beam computed tomography (CBCT) [10,20]. Panoramic radiography, or orthopantomography (OPG), is the imaging method widely used in dentistry [21,22] and has good clinical applicability in the field of implant surgery [23]. The advantages of this imaging method include a low radiation dose, low cost, and exposure of bone changes, which allow analysis of trabecular bone [24,25].

In addition to radiological evaluation, a method that helps in the quantitative and qualitative assessment of bone structure is represented by fractal analysis (FA) [10,26]. The concept of fractal analysis was first introduced in 1980 for use in many branches of science [7,27,28,29,30,31,32,33]. This concept has also been adopted in dentistry to analyze medical imaging patterns [7,27], and it is considered a diagnostic tool that can objectively assess the complexity of structures [7,34,35,36]. Fractal analysis is a mathematical technique, and the numerical value obtained is known as the fractal dimension (FD) [37]. Fractal dimension can be used in OPG as a descriptor of the complex architecture of trabecular bone, with good clinical applicability and serving as a distinctive parameter for determining bone tissue density [7,22,38,39,40,41]. Trabecular bone exhibits properties such as self-similarity and the absence of a well-defined scale, which allows for the analysis of fractal dimension [42,43,44]. Studies in the literature have shown a correlation between trabecular bone architecture and fractal dimension [20], with a higher fractal dimension directly associated with a more complex trabecular pattern [10,22]. Both cortical and trabecular bone exhibit a fractal structure, and FD analysis of dental radiographs serves as a reference test for bone mineral density [45,46].

The microarchitecture of the alveolar bone is very important for ensuring the stability of future implant-prosthetic restorations [11,43]. This method of analyzing the trabecular pattern has been successfully applied to OPG images, with numerous studies demonstrating a correlation between fractal dimension and bone mass, allowing the detection of variations in trabecular bone density and bone tissue demineralization [8,26,47]. Fractal analysis is a procedure for processing data in image form, in which the information is stored as numbers [48]. The study by White and Rudolph [49] described the steps for analyzing trabecular bone using Image J. After preparing the radiological images, different methods of calculating the fractal dimension can be used [31]. The box-counting method is the most widely used for determining fractal dimension and has the advantage of assessing changes in trabecular bone [42,50]. Also, the selection of the region of interest (ROI) is an essential criterion for determining FD values, as the diversity of ROIs can lead to differences in FD values [31].

The present study aimed to analyze changes in the alveolar bone following tooth extraction, using fractal analysis of OPG images, comparing values obtained before the surgical intervention with those obtained 3 months after it, both in the case of natural healing and in the case of immediate placement of dental implants. The null hypothesis was that the post-extraction values obtained in the areas studied by fractal analysis at 3 months show no differences between natural healing and immediate placement of dental implants, and do not differ from the values obtained from the initial measurements.

## 2. Materials and Methods

### 2.1. Study Design

In this retrospective study, fractal analysis was performed on OPG images (before tooth extraction and 3 months after the surgical procedure) extracted from the medical files of patients who underwent dental extractions in the maxillary and mandibular molar areas and in whom either natural healing of the post-extraction alveoli was expected or dental implants were placed immediately after tooth extraction. The patients included in the study were selected from the Department of Oral Rehabilitation of the University of Medicine and Pharmacy of Craiova, who presented between January 2023 and January 2025 for the establishment of an implant-prosthetic treatment plan. The study protocol was approved by the Ethics and Academic Integrity Committee of the University of Medicine and Pharmacy of Craiova (Approval number 231/28.11.2022). All patients included in the study accepted the treatment plan, and surgical interventions were performed after they understood and signed the written informed consent. Subjects were treated in full compliance with applicable ethical principles, including the World Medical Association Declaration of Helsinki (2013 version).

### 2.2. Patient Selection

The study group consisted only of non-smoking, systemically healthy patients who had maxillary or mandibular molars that were to be extracted as part of the implant-prosthetic rehabilitation treatment plan.

Inclusion criteria:

- Patients over 18 years of age.
- Patients with maxillary or mandibular molars requiring extraction, which can no longer be restored.
- Systemically healthy patients, classified in ASA I and ASA II classes.
- Compliant and cooperative patients.
- Patients who requested implant-prosthetic rehabilitation.

Exclusion criteria:

- Patients with systemic diseases, such as severe hypertension, diabetes, renal disease, or hepatic disease.
- Patients under treatment with anticoagulants, systemic steroids, or systemic bisphosphonates.
- Patients who smoke (>10 cigarettes per day), are alcoholics or drug addicts.
- Pregnant or lactating patients.
- Patients who have previously undergone radiotherapy to the surgical area.
- Patients with poor oral hygiene.
- Patients who have had an acute tooth infection that required extraction.

### 2.3. Case Evaluation

The data extracted from the medical files of the patients included in the study included information obtained following assessment of general health status, intraoral and extraoral clinical examination, OPG radiological examination, and laboratory examinations. Also, all information regarding diagnoses, individualized treatment plans for each patient, the timing of tooth extractions, the type of dental implants, incidents, and complications that occurred during treatment was collected. Data regarding post-interventional consultations at 1 week, 1 month, and 3 months were also collected. All control radiographs were analyzed; for the study, those performed before tooth extraction and 3 months after the surgical intervention were used to observe bone changes following the procedure. The data obtained were used to divide the cases into two groups: the control group, in which natural healing was expected, and the study group, in which implants were inserted immediately post-extraction, at the patient’s choice.

### 2.4. Radiographic Evaluation

Radiological evaluation was performed on orthopantomograms obtained before the surgical procedure and 3 months after it, using the Fona XPan 3D Plus device (FONA Dental s.r.o., Milano, Italy).

### 2.5. Fractal Analysis

Fractal analysis was performed on digital OPG radiological images (stored in tiff format), using Image J software (National Institutes of Health (NIH), Bethesda, MD, USA, version 1.54j) with the FracLac plugin, used for surface area analysis based on the box counting method for estimating the fractal dimension (FD). This method is applied to binary images and uses multiple boxes (or grids) of different sizes to represent and cover a complex shape, counting the number of occupied boxes at each scale.

One single dentomaxillofacial radiologist performed all OPG measurements. Calibration was performed on a separate set of 30% randomly selected radiographs, 2 weeks after the initial assessment. Intra-observer reliability was assessed using the intraclass correlation coefficient (ICC), calculated via a two-way mixed-effects model for absolute agreement, resulting in an excellent agreement (0.939).

To accurately analyze the data from the digital radiographic images taken before and after the surgical procedure, stable anatomical landmarks were chosen as reference points: the maxillary sinus for the maxilla and the mandibular canal for the mandible. Subsequently, a region of interest (ROI) of 50 × 50 pixels was selected in the maxillary and mandibular molar areas, following the anatomical landmarks chosen as reference points, avoiding their overlap, and the fractal index was calculated for the image within it. The same region of interest was chosen for each radiological image. Thus, in the maxilla, the area of interest was bordered superiorly by the contour of the maxillary sinus. In the mandible, the area of interest was bordered inferiorly by the contour of the mandibular canal. Regarding the distance from neighboring teeth, a 2 mm distance was chosen, as this is the minimum required for inserting dental implants.

**Figure 1:**
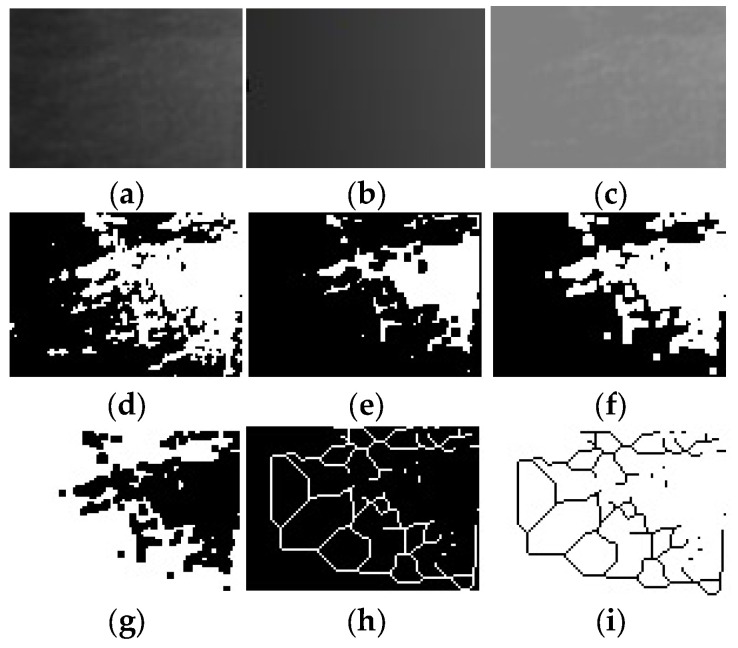
FD analysis protocol: (**a**) cropping and duplicating the ROI, (**b**) applying a Gaussian filter, (**c**) adding a mathematical value of 128, (**d**) binarization, (**e**) eroding, (**f**) dilating, (**g**) inverting, (**h**) skeletonization, and (**i**) inverting.

The analysis protocol included the following sequence of steps: cutting and duplicating the ROI (Figure 1a), applying a Gaussian filter with sigma = 35 (Figure 1b), subtracting duplicate images and adding a mathematical value of 128 (Figure 1c), binarization (Figure 1d), erosion (Figure 1e), dilation (Figure 1f), inversion (Figure 1g), skeletonization (Figure 1h) and finally inversion of the image once again (Figure 1i).

Finally, the differences between the measurements obtained from the fractal analysis of the initial radiological images and those taken three months after surgery were calculated.

### 2.6. Statistical Analysis

The data collected from the patients’ medical charts were initially processed using Microsoft Excel 365 (San Francisco, CA, USA). The software application SPSS (Statistical Package for Social Sciences) software, version 26 (SPSS Inc., Armonk, NY, USA) was used to statistically analyze the data, both in a descriptive and inferential manner. The study parameters were expressed as frequency distributions and associated percentages, for nominal and ordinal parameters, while continuous variables were defined as “mean ± standard deviation (SD)” or potentially median values (in association with *p*-values, for non-normally distributed values). Intra-rater reliability for FD measurements was evaluated using the intraclass correlation coefficient (ICC). Normality was assessed based on the Shapiro–Wilk test. In accordance, comparisons between groups for continuous variables were performed using either the Mann–Whitney U test, the paired *t*-test, or the two-way ANOVA. Nominal parameters were assessed using the Chi-Square test. Also, a multiple regression model was developed to determine the effects of several parameters on predicting the FD variation after 3 months from the beginning of the study. This model included the presence of an implant following the extraction, age groups, gender, residence, maxillary/mandible location, and the molar type. *p*-values smaller than 0.05 represented statistically significant results.

## 3. Results

### 3.1. Baseline Data

The study group included 91 patients, 45 female and 46 male, aged 27–78 years, with a mean age of 50.48 ± 11.99 years. The patients came from both urban and rural areas, with the majority residing in urban areas (68 patients, 74.72% of the entire study group).

The patients were divided into two subgroups: the control group (No implant), consisting of 48 patients who were expected to heal naturally after dental extractions, and the study group (Implant), composed of 43 patients who opted for immediate post-extraction dental implant placement (SuperLine, Dentium Co., Ltd., Suwon, Republic of Korea). These two groups were demographically balanced, with no statistically significant differences in gender, age, or residency between groups (*p* > 0.05; Table 1).

The majority of extractions (52, 57.14%) were performed in the maxilla and were distributed similarly between the two study groups, with no statistically significant differences in extraction location between groups (*p* = 0.856; Table 2). More than half of the extractions were performed at the level of the first molar, followed by the second molar and the third molar. For the first and second molars, extractions were similarly distributed in both groups, with percentages close to 50%. In contrast, the third molar was extracted only in the control group, yielding a borderline *p*-value but no statistical significance (Table 2).

### 3.2. Fractal Analysis Results

Table 3 presents the minimum, maximum, mean ± SD, and median values for all FA parameters obtained using the box-counting method, which uses multiple boxes (or grids) of different sizes to cover the complex bone structure. The differences in bone structure were quantified as the values obtained by subtracting the measurements after extractions from those before extractions. Thus, higher FD values at follow-up (3 months) reflected greater bone remodeling, as FD values are directly proportional to the complexity of bone structure. Therefore, a positive difference would reflect bone resorption, as the bone’s complexity after 3 months would be lower than before extraction. A negative difference would reflect bone healing. Also, higher FD variations would represent accelerated processes, while lower FD variations would represent slower processes, for both healing and resorption.

For each parameter computed within the fractal analysis, Table 4 indicates the number of positive differences (thus better bone structures after the extraction), as well as the number of negative differences (therefore poorer bone structures). According to this table, there is a slight predominance of negative differences in the evolution of patients with an implant compared with those without an implant (Table 4).

For all patients included in the study subgroup Implant, there are more negative differences; the presence of the implant seems to favor bone healing during the first three months following extraction and implant insertion. In the other study subgroup, No Implant, patients exhibited both bone resorption and optimal healing, with a distribution that was pretty similar.

For both groups, the FD values at the 3-month follow-up indicate a decrease compared to the initial values; the difference between the two time points is not statistically significant (*p* > 0.05; Table 5).

A two-way ANOVA was conducted to examine the effects of the clinical and demographical parameters acquired in the study, in association with the absence or presence of an implant following the extraction, on the FD variation between the initial moment of the study and the 3-month follow-up. Residual analysis was performed to test for the assumptions of the two-way ANOVA. Outliers were assessed by inspection of a boxplot, normality was assessed using Shapiro–Wilk’s normality test for each cell of the design, and homogeneity of variances was assessed by Levene’s test. All pairwise comparisons were run, with reported 95% confidence intervals and *p*-values that are Bonferroni-adjusted.

**Figure 2:**
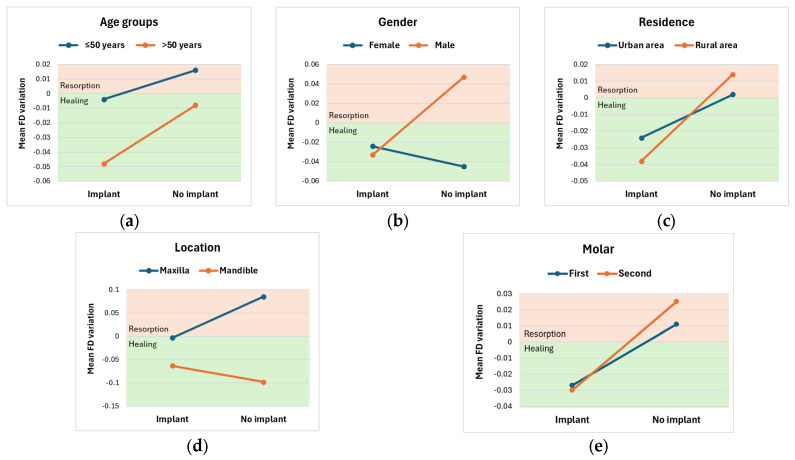
FD variation according to the presence/absence of an implant and: (**a**) age groups; (**b**) gender; (**c**) residence; (**d**) location; (**e**) molar.

Following analysis of the patients’ ages, it appears that both patients below and above 50 years old exhibit greater FD variations, reflecting a faster bone-healing process in the presence of an implant, which is more accelerated in elderly patients (Figure 2a). Still, the interaction between the Implant and No Implant lots and age groups on FD variation was not statistically significant, F(1, 97) = 0.070, *p* = 0.792, partial η^2^ = 0.001. Therefore, an analysis of the main effect for study subgroups was performed, which indicated that the main effect was not statistically significant, F(1, 97) = 0.623, *p* = 0.432, partial η^2^ = 0.007. The unweighted marginal means of the FD variation for patients with an implant and with no implant were −0.026 ± 0.028 and 0.004 ± 0.026, respectively. Similarly, an analysis of the main effect for age groups indicated that it was not statistically significant, F(1, 87) = 0.802, *p* = 0.373, partial η^2^ = 0.009. The unweighted marginal means of FD variation for patients aged < 50 and above 50 were −0.006 ± 0.027 and −0.028 ± 0.026, respectively.

According to Table 6, gender influences patients’ evolution; evolution patterns differ. Patients of both genders with implants exhibit mostly negative FD variations, indicating bone healing, and females exhibit smaller FD variations than men. However, males without implants exhibit predominantly bone resorption (positive variations), while females without implants exhibit accelerated bone healing (high negative variations) (Figure 2b). Overall, there was no statistically significant interaction between gender and implant presence on FD variation, F(1, 87) = 1.815, *p* = 0.181, partial η^2^ = 0.020. There was no statistically significant main effect of implant presence on FD variation, F(1, 87) = 0.622, *p* = 0.433, partial η^2^ = 0.007. The unweighted marginal means of FD variation for patients with an implant and with no implant were −0.028 ± 0.027 and 0.001 ± 0.026, respectively. Also, there was no statistically significant main effect of gender on FD variation, F(1, 87) = 1.228, *p* = 0.271, partial η^2^ = 0.014. The unweighted marginal means of FD variation for females and males were −0.034 ± 0.026 and 0.007 ± 0.026, respectively.

Patients with both urban and rural residence exhibit better bone healing processes in the presence of an implant, as well as bone resorption after extraction only, so somewhat similar behaviors, with no statistically significant interaction between residence and implant lot for FD variation, F(1, 87) = 0.089, *p* = 0.766, partial η^2^ = 0.001 (Figure 2c). There was no statistically significant main effect of implant presence on FD variation, F(1, 87) = 0.800, *p* = 0.373, partial η^2^ = 0.009. The unweighted marginal means of FD variation for implant and no implant patients were −0.031 ± 0.030 and 0.008 ± 0.032, respectively. There was no statistically significant main effect of residence on FD variation, either, F(1, 87) < 0.0005, *p* = 0.982, partial η^2^ < 0.0005. The unweighted marginal means of FD variation for urban and rural patients were 0.001 ± 0.044 and −0.001 ± 0.044, respectively.

In patients undergoing maxillary and mandibular extractions, FD variation patterns differ between groups. Even if there was no statistically significant interaction between location and implant presence for the FD Variation, F(1, 87) = 3.061, *p* = 0.084, partial η^2^ = 0.034, the *p* value was only a little higher than the threshold value. Patients with implants, regardless of extraction site, showed mean negative FD variations, indicating bone-healing processes, more accelerated in the mandible than in the maxilla. For patients without an implant, there is a clear difference: the mandible shows a trend toward accelerated healing, whereas patients with maxillary extractions tend to exhibit bone resorption (Figure 2d). Independently, the implant’s presence had no statistically significant main effect on FD variation, F(1, 87) = 0.558, *p* = 0.457, partial η^2^ = 0.005. The unweighted marginal means of FD variation for implant and no implant patients were −0.033 ± 0.026 and −0.007 ± 0.024, respectively. The presence of the implant causes the jawbone to behave similarly to the mandible bone. On the other hand, also independently considered, the location had a statistically significant main effect on FD variation, F(1, 87) = 11.811, *p* = 0.001, partial η^2^ = 0.120. The marginal means for FD variation were 0.041 ± 0.012 for maxillary and −0.080 ± 0.027 for mandible, with a statistically significant mean difference of 0.121 (95% CI, 0.051 to 0.191), *p* = 0.001.

Following analysis of the molar type, it appears that patients with various extracted molars exhibit similar behavior, with mostly bone healing in the presence of an implant, compared to bone resorption otherwise (Figure 2e). There was no statistically significant interaction between molar type and implant lot for FD variation, F(1, 86) = 0.040, *p* = 0.841, partial η^2^ < 0.0005. There was no statistically significant main effect of lot implant on FD variation, F(1, 86) = 1.252, *p* = 0.266, partial η^2^ = 0.014. The unweighted marginal means of FD variation for implant and no implant patients were −0.029 ± 0.029 and −0.012 ± 0.031, respectively. Similarly, there was no statistically significant main effect of molar type on FD variation, F(1, 86) = 0.628, *p* = 0.536, partial η^2^ = 0.014. The unweighted marginal means of FD variation for molar 1, molar 2, and molar 3 were −0.008 ± 0.024, −0.002 ± 0.034, and −0.071 ± 0.073, respectively.

Overall, Table 6 compares FD variation by implant presence/absence, for each of the five parameters defined in the rows.

The presence of the implant influences the FD values on OPG, which are the bone healing pattern, so that bone resorption is reduced compared to that in the case of natural healing.

A multiple regression was run to predict FD variation after 3 months from extraction, based on the presence/absence of an implant, gender, age group, residence, location, and molar type. There was linearity as assessed by partial regression plots and a plot of studentized residuals against the predicted values. There was independence of residuals, as evaluated by a Durbin–Watson statistic of 1.631. There was homoscedasticity, as assessed by visual inspection of a plot of studentized residuals versus unstandardized predicted values. There was no evidence of multicollinearity, as evaluated by tolerance values greater than 0.1. There were no studentized deleted residuals greater than ±3.5 standard deviations, no leverage values greater than 0.2, and values for Cook’s distance above 1. The assumption of normality was met, as assessed by a Q-Q Plot. The multiple regression model statistically significantly predicted the FD variation after 3 months, F(6, 84) = 2.930, *p* = 0.012, adj. R^2^ = 0.114.

According to Table 7, the only significant parameter within the model was the location, thus the predicted FD variation for extraction at the mandible level is, on average, 0.130 smaller than the predicted FD variation for extractions at the maxillary level (with all values of all other independent variables being held constant), confirming the results of the univariate analysis emphasizing better bone healing (and a smaller bone resorption) for the mandible compared to the maxillary.

## 4. Discussion

The present study addresses two current issues on the agenda of implantology researchers: how to prevent post-extraction bone resorption [51], especially in molars, and whether immediate placement of dental implants influences bone healing [52]. Using fractal analysis of OPG images obtained pre-extraction and 3 months after tooth extraction, a comparison between natural bone healing and bone healing in the presence of a dental implant placed immediately post-extraction, without loading, showed a better outcome in cases with dental implants. Bone healing was better in the mandible than in the maxilla, which is interesting, given that the mandible has denser and less vascularized bone than the maxilla [53].

In recent years, implant-prosthetic treatment has become a standard method of rehabilitation for patients with edentulous areas. To ensure the success of this type of treatment, it is essential to objectively assess the quality and quantity of post-extraction alveolar bone [54,55,56,57,58,59,60,61,62], as well as to control the resorption and remodeling phenomena occurring during the healing process [63,64,65]. The primary stability of the implant and the osseointegration period depend on factors such as an uncomplicated surgical procedure, implant surface, and its characteristics [7].

The immediate placement technique for dental implants has the advantage of preserving anatomical characteristics by reducing bone resorption, maintaining alveolar bone volume, and supporting the surrounding soft tissues, factors that are particularly important for achieving successful treatment [66]. To comprehensively evaluate the quality of the jaw bones and the changes in the post-extraction alveolar bone, in addition to histological analysis, it is necessary to assess individual parameters through clinical examinations and radiological analyses [8]. In this context, the study by Sanchez et al. made a significant contribution to this field by applying fractal analysis to identify variations in fractal dimension before and after dental implant placement, highlighting fractal analysis as a tool for evaluating osseointegration [28].

The evaluation of changes in bone structure primarily relies on trabecular bone, which has higher metabolic activity than cortical bone [67]. Numerous studies in the literature have analyzed the trabecular structure of alveolar bone using dental radiographs. Orthopantomography is an imaging technique widely used in dental practice, as it is cost-effective and easily accessible for evaluating bone mineral density changes [68,69]. In the study conducted by Parekh et al., the trabecular architecture of the maxillary bones was examined on OPG images before and after orthodontic treatment. Their results showed statistically significant changes in the trabeculae of the lower anterior teeth, indicating bone resorption. They concluded that fractal dimension analysis is a valuable method for studying the trabecular pattern on two-dimensional images [48]. The study conducted by Magat et al. compared the FD analysis of CBCT and OPG images. They found that orthopantomography is more advantageous for examining trabecular bone due to its lower radiation dose and higher image resolution [70].

In this retrospective study, changes in alveolar bone following tooth extractions were analyzed, comparing natural healing with immediate implant placement. Fractal analysis of OPG images was used to compare values obtained before surgery and 3 months after surgery. The 3-month period was chosen because the highest rate of bone resorption occurs in the first 3 months after tooth extraction, consistent with other studies [63,64,65,71]. In the review conducted by Mishra et al., an attempt was made to provide scientific evidence to determine whether FD can be used to assess implant stability through quantitative measurements of peri-implant bone density. The results of the included studies led to the conclusion that FD analysis can be used as a viable and easily accessible method for bone quantification in implant treatment [72]. In the study conducted by Öztürk et al., comparative analyses performed before and after implant placement revealed a significant increase in FD values 3 months after dental implant placement [73].

In the study by Koh et al., it was demonstrated that FD analysis of two-dimensional orthopantomographic images is a useful method in the mandibular premolar region, suggesting that the trabecular pattern can be clearly observed even in dense bone [74]. Based on the same reasoning, in the study conducted by Soylu et al., FD evaluation was performed on panoramic images of the mandibular premolar and molar regions [7]. Given that the literature demonstrates that the most significant bone resorption occurs in the molar areas [5,7], the dental extractions included in the present study were also performed in the maxillary and mandibular molar regions. The same surgical protocol was followed for all patients, and no signs of inflammation or post-extraction complications were observed. There was homogeneity in terms of patient gender, but most patients came from urban areas. Most extractions were performed in the maxilla and especially in the first molar.

Fractal analysis has been used to evaluate bone structure in various conditions, including periodontal disease, temporomandibular joint disorders, and bruxism. Also, numerous techniques have been developed to assess post-extraction alveolar bone [10]. Fractal analysis is considered an effective method for analyzing the microarchitecture of the maxillary bones [75,76,77,78,79,80], being an accurate, non-invasive, accessible, easy to perform, economical, and efficient method that can be performed even on conventional radiographs [11,19,26,31,47,81]. FD analysis of OPG images is a reliable method because the trabecular pattern can be well visualized in dense bone. The shades of gray represent different densities of anatomical structures; dense bone appears lighter, and spongy tissue appears darker [74]. Compared to other medical imaging methods, such as CBCT or micro-CT, the advantages of two-dimensional radiographs include high resolution, low cost, non-invasiveness, and low radiation dose. Disadvantages include risk of overlapping and blurring of some structures, low precision, potential image distortion, reduced image contrast that can affect software during digital processing, and inability to capture fine details [31,42,82]. Therefore, in the present study, fractal analysis was performed on OPG images of the maxillary and mandibular molars. It has been reported that trabecular bone has a higher renewal rate than cortical bone, due to its higher surface-to-volume ratio, which responds more quickly to metabolic and hormonal changes [35]. OPG images have been used to assess the FD and lacunarity of the mandibular bone in patients who underwent radiotherapy. Barcelos et al. showed that the mean FD and lacunarity values in the study and control groups were similar and did not differ significantly [25]. Palma et al. obtained a slight reduction in FD of the mandibular bone after radiotherapy (RT) (1.3 ± 0.1) compared to FD before RT (1.4 ± 0.1) [24]. In their study, Sevimay et al. aimed to evaluate potential changes in mandibular trabecular bone structure associated with the use of antiresorptive drugs in oncology patients, using fractal analysis on OPG images. Their results indicated that antiresorptive therapy significantly affected trabecular bone structure, with notable differences in FD values among the osteoporotic, oncological, and control groups. The mean FD values in the molar region of patients with osteoporosis were significantly lower (1.26 ± 0.18) than those of the control group (1.32 ± 0.07) [83].

Mangano et al. found that osseointegration is influenced by bone metabolism, which may vary by gender [84]. In contrast, Chen et al. found that although ISQ scores were lower in females than in males, the difference was not statistically significant [85]. Akkoca et al. observed statistically significant differences between females and males in terms of fractal analysis [8]. Soylu et al. did not observe statistically significant differences between genders [7]. Srinivas et al. designed a study to investigate the application of fractal analysis to analyze the trabecular bone pattern in both genders, using OPG images. Their study showed that females have a lower FD value than their male counterparts, suggesting a difference in trabecular pattern between the two genders, which is explained by the fact that females may have a less dense bone pattern than males [86].

Most studies in the literature that performed fractal analysis of OPG images aimed to measure bone mineral density. Most of the time, the increase in fractal dimension was associated with newly formed bone tissue and with a complex bone structure [35,81]. Also, in other studies, higher FD values were considered to indicate a more complex, denser bone structure, and lower FD values were associated with bone loss and resorption [68]. On the other hand, in other studies in the literature, it was reported that when bone mineral density decreases, the trabecular bone structure becomes more complex, being directly proportional to the number of fractals [31]. Sansare et al. reported a significantly higher FD value after dental implant insertion than the initial FD value, due to increased bone quantity around the implants [87]. In the study conducted by Soylu et al., FD analysis was performed at four time points: before dental implant insertion (T0), 1 week (T1), 1 month (T2), and 2 months (T3) after surgery. FD values in T1 were significantly lower compared to T0, decreasing in the first week, and then gradually increasing, and at two months, FD values were significantly increased compared to T0 values [7]. In the present study, differences in bone structure were quantified by subtracting the values obtained from post-extraction measurements from the values obtained from pre-extraction measurements. Thus, higher FD values obtained at 3 months post-extraction reflected greater bone remodeling.

In addition, the resolution and format of the images are particularly important factors for making accurate assessments [37]. In the study conducted by Toghyani et al., the fractal dimension (FD) was compared across different image formats, resolutions, and compression levels, and they found that FD increased with increasing resolution [38]. Also, in the study by Yasar et al., it was found that the FD value was higher for TIFF than for JPEG format images [88]. Thus, in our study, TIFF-format radiological images were used, obtained with the same radiological device in all cases.

In the study by Aktuna Belgin et al., no statistically significant differences were observed between genders. Still, individual differences regarding bone size, bone metabolism, and patient medical history may influence the results [42]. In agreement, the results of the present study, as well as those of studies in the specialized literature, showed that variables such as gender, age, environment of origin, and molar type do not significantly influence the bone pattern observed in fractally analyzed radiological images.

The study conducted by Akkoca et al. aimed to use OPG images to compare alveolar bone density in the peri-implant area using fractal analysis, both before and after osseointegration, for dental implants. The study group’s analysis demonstrated a correlation between pre- and post-implantation values, suggesting changes over time, especially in female patients. In their study, the loading time of dental implants was set at 4 months after insertion, due to an increase in fractal dimension. Statistically significant differences were also identified in fractal analysis between the maxilla and mandible, in both cases demonstrating a significant increase in bone microstructure around the implant and in the number of bone trabeculae after osseointegration [8].

The study by Singh et al. showed better bone healing in the crestal region (crestal bone level) with delayed implant placement, with or without bone graft, compared with immediate implant placement. Conversely, immediate implant placement offers several advantages: reduced surgical time and sessions, reduced costs, reduced edentulism time, and alveolar ridge preservation [12]. In the study by Xu et al., dental implants were placed into the sockets immediately after extraction, and the results showed that they provided advantages such as less trauma, complete use of the gingiva, and a well-preserved alveolar ridge morphology compared with natural healing and delayed placement of dental implants [89]. The results of this study showed that in the case of the study group in which dental implants were placed immediately after tooth extraction, all patients experienced a faster healing process, the bone having a better bone density, without statistically significant differences in terms of location, and in the case of the control group in which natural healing was expected, all patients showed a balance between bone healing and bone resorption, with differences between the maxilla/mandible, the mandible showing a faster healing process than the maxilla. According to ITI consensus recommendations [90], treatment planning for implant-prosthetic therapy should begin before dental extraction. All cases should be carefully evaluated, and immediate implant placement should be considered whenever predictable outcomes can be fulfilled. Due to the risk of alveolar ridge resorption, late implant placement is considered the least desirable option; in this case, alveolar ridge preservation methods are indicated.

Limitations of the study include a smaller number of cases in sublots and the spatial limitations of radiographic images, which are not three-dimensional and imply anatomical structures’ superposition. Orthopantomography was chosen for this study because it is a radiological investigation that can be repeated more often than CBCT, the second being more invasive due to a higher radiation dose. Although fractal analysis can be a useful exploratory tool, its scientific validity depends largely on methodological rigor. Another limitation concerns the comparison that was made on patients who had benefited from different treatments, but the groups were chosen this way because the patients either requested implants or requested natural healing.

## 5. Conclusions

Fractal analysis highlighted minor differences in bone-healing patterns between the dental implant area and simple post-extraction healing at 3-month follow-up. In the case of peri-implant bone, healing after 3 months following extraction and implant insertion was characterized by minimal reductions in FD values compared to simple healing, indicating that bone healing in the presence of the implant was similar to natural healing. The fractal dimension values obtained showed statistically significant differences between the maxillary and mandibular locations, associated with the absence of the dental implant. In this case, in the presence of the implant, FD values were similar in the maxilla and mandible, indicating that the implant improves healing in the maxilla.

## Figures and Tables

**Table 1:** Demographic characteristics of patients included in the study.

Study Variable	Category	Subgroup		Total	*p*
		Implant (%)	No Implant (%)		
Patients	-	43 patients	48 patients	91 patients	
	F	23 (51.11%)	22 (48.89%)	45 (100%)	
Gender		53.49%	45.83%		0.466 *
	M	20 (43.48%)	26 (56.52%)	46 (100%)	
		46.51%	54.17%		
Age (years old)	Median	53.00	50.00	-	0.578 **
	Mean ± SD	51.23 ± 11.318	49.81 ± 12.640		
	≤50	19 (43.18%)	25 (56.82%)	44 (100%)	
Age groups		44.19%	52.08%		0.452 *
(years old)	>50	24 (51.06%)	23 (48.94%)	47 (100%)	
		55.81%	47.92%		
Residence	Urban	30 (44.12%)	38 (55.88%)	68 (100%)	
		69.77%	79.17%		0.303 *
	Rural	13 (56.52%)	10 (43.48%)	23 (100%)	
		30.23%	20.83%		

* Chi-Square test. ** Mann–Whitney U test. Cells emphasized in light grey contribute to the sum of values by column.

**Table 2:** Characteristics of patients included in the study, relative to demographic characteristics.

Study Variable	Category	Subgroup		Total	*p*
		Implant (%)	No Implant (%)		
Patients	-	43 patients	48 patients	91 patients	
Location	Maxilla	25 (48.08%)	27 (51.92%)	52 (100%)	
		58.14%	56.25%		0.856 *
	Mandible	18 (46.15%)	21 (53.85%)	39 (100%)	
		41.86%	43.75%		
Molar	First molar	29 (50.88%)	28 (49.12%)	57 (100%)	
		67.44%	58.33%		
	Second molar	14 (50%)	14 (50%)	28 (100%)	0.056 *
		32.56%	29.17%		
	Third molar	0 (0%)	6 (100%)	6 (100%)	
		0%	12.5%		

* Chi-Square test. Cells emphasized in light grey contribute to the sum of values by column.

**Table 3:** Average values of fractal analysis—before and 3 months after tooth extraction.

Group	FA Parameters		C2	C3	C4	C6	C8	C12	C16	C32	C64	FD
		Min	45	32	27	17	13	11	8	4	1	1.021
	Before	Max	175	120	89	59	41	23	16	4	1	1.486
		Median	125	85	65	43	31	17	11	4	1	1.378
		Mean ± SD	120.884 ± 27.325	84.86 ± 18.821	63.721 ± 13.222	42.442 ± 8.419	29.651 ± 5.818	17.302 ± 2.474	11 ± 1.877	4 ± 0	1 ± 0	1.356 ± 0.09
		Min	51	32	27	18	13	8	5	2	1	1.07
	After	Max	170	114	94	60	41	22	15	4	1	1.481
Implant		Median	113	78	61	40	30	17	11	4	1	1.338
		Mean ± SD	113.14 ± 32.392	78.442 ± 21.208	60.023 ± 16.133	39.512 ± 10.051	28.977 ± 6.926	16.721 ± 3.568	10.791 ± 2.294	3.907 ± 0.426	1 ± 0	1.328 ± 0.101
		Min	−77	−54	−38	−23	−20	−9	−7	−2	0	−0.297
	Diff	Max	71	41	31	23	13	6	5	0	0	0.188
		Median	−7	−8	−4	−4	−1	0	0	0	0	−0.02
		Mean ± SD	−7.744 ± 34.6	−6.419 ± 22.398	−3.698 ± 16.683	−2.93 ± 10.425	−0.674 ± 7.396	−0.581 ± 3.54	−0.209 ± 2.474	−0.093 ± 0.426	0 ± 0	−0.028 ± 0.112
		Min	7	5	3	3	1	1	1	1	1	0.603
	Before	Max	164	116	85	55	37	22	14	4	1	1.468
		Median	95.5	67.5	52	36	25.5	14.5	9	4	1	1.283
		Mean ± SD	92.521 ± 34.798	64.75 ± 23.736	49.042 ± 17.718	32.583 ± 11.276	23.646 ± 7.821	13.771 ± 4.299	9.146 ± 2.423	3.625 ± 0.789	1 ± 0	1.256 ± 0.156
		Min	13	11	8	5	4	3	2	1	1	0.833
No	After	Max	160	107	87	56	42	24	15	4	1	1.457
implant		Median	91	63	50	33	24	14.5	9	4	1	1.268
		Mean ± SD	93.563 ± 32.513	65.167 ± 22.153	50.104 ± 16.74	33.479 ± 10.995	24.5 ± 7.663	14.646 ± 4.339	9.708 ± 2.713	3.729 ± 0.676	1 ± 0	1.261 ± 0.131
		Min	−107	−75	−54	−36	−26	−13	−9	−3	0	−0.532
	Diff	Max	125	78	60	42	27	17	12	3	0	0.697
		Median	−0.5	1.5	−0.5	1.5	0	1	1	0	0	−0.0005
		Mean ± SD	3.271 ± 47.727	0.417 ± 35.152	1.063 ± 25.922	0.896 ± 17.211	0.854 ± 11.7	0.875 ± 6.447	0.563 ± 4.01	0.104 ± 1.134	0 ± 0	0.005 ± 0.221

**Table 4:** Differences analysis for the FA parameters.

FA Parameter	C2	C3	C4	C6	C8	C12	C16	C32	C64	FD
**Implant**										
Positive diff	16	17	17	17	17	20	18	0	0	16
Negative diff	27	26	26	26	26	23	25	43	43	27
**No implant**										
Positive diff	23	24	23	26	24	29	26	11	0	24
Negative diff	25	24	25	22	24	19	22	37	48	24

**Table 5:** Bone evolution reflected by the FD values, by study lot.

Time Period	FD Values					
	Implant		No Implant		Variation	
	Median	Mean ± SD	Median	Mean ± SD	Median	Mean ± SD
Before	1.378	1.356 ± 0.090	1.283	1.256 ± 0.156	−0.020	−0.028 ± 0.112
After	1.338	1.328 ± 0.101	1.268	1.261 ± 0.131	−0.005	0.005 ± 0.221
*p* *	0.158		0.943		0.443	

* Paired *t*-test.

**Table 6:** Bone evolution reflected in FD values across age groups, gender, residence, extraction location, and molar type.

Study Variables/ Categories	Bone Evolution—FD Values Variation (Mean ± SD)			F	*p* *	Partial η^2^
	Individual Significance	Implant	No Implant			
Age groups		*p* = 0.432 (subgroup)				
≤50 years	*p* = 0.373	−0.004 ± 0.117	0.016 ± 0.193	0.070	0.792	0.001
>50 years		−0.048 ± 0.105	−0.008 ± 0.252			
Gender		*p* = 0.433 (subgroup)				
Female	*p* = 0.271	−0.024 ± 0.110	−0.045 ± 0.132	1.815	0.181	0.020
Male		−0.033 ± 0.116	0.047 ± 0.271			
Residence		*p* = 0.373 (subgroup)				
Urban	*p* = 0.982	−0.024 ± 0.096	0.002 ± 0.187	0.089	0.766	0.001
Rural		−0.038 ± 0.145	0.014 ± 0.335			
Location		*p* = 0.558 (subgroup)				
Maxilla	*p* = 0.001 ^#^	−0.003 ± 0.090	0.085 ± 0.192	3.061	0.084	0.034
Mandible		−0.063 ± 0.131	−0.098 ± 0.218			
Molar		*p* = 0.266 (subgroup)				
First	*p* = 0.536	−0.027 ± 0.120	0.011 ± 0.243	0.040	0.841	<0.0005
Second		−0.030 ± 0.096	0.025 ± 0.198			
Third		-	−0.071 ± 0.176			

* Two-way ANOVA. ^#^ Statistically significant.

**Table 7:** Multiple regression model—linear coefficients.

Parameter	B	t	Sig	CI Interval *	
				Lower	Upper
Subgroup (Implant/No Implant)	0.032	0.891	0.375	−0.040	0.104
Gender (Female/Male)	0.052	1.427	0.157	−0.020	0.124
Age groups (≤50, >50 years)	−0.051	−1.392	0.168	−0.124	0.022
Residence (Urban/Rural)	−0.010	−0.227	0.821	−0.093	0.074
Location (Maxillary/Mandible)	−0.130	−3.647	<0.0005 ^#^	−0.201	−0.059
Molar type (M1, M2, M3)	−0.027	−0.874	0.385	−0.087	0.034

^#^ Statistically significant values. * 95% confidence interval.

## Data Availability

The data presented in this study are available on request from the corresponding author due to privacy, legal, and ethical restrictions.

## Abbreviations

The following abbreviations are used in this manuscript:

OPG	Orthopantomography
CBCT	Cone Beam Computed Tomography
FA	Fractal Analysis
FD	Fractal Dimension
ROI	Region of Interest
ASA	American Society of Anesthesiologists
NIH	National Institutes Of Health
SPSS	Statistical Package for Social Sciences
SD	Standard Deviation
ISQ	Implant Stability Quotient
